# Anaphylaxie durch Epoxidharz während einer zahnärztlichen Behandlung

**DOI:** 10.1111/ddg.15850_g

**Published:** 2026-01-14

**Authors:** Julia Oberschmied, Elsbeth Oestmann, Margitta Worm

**Affiliations:** ^1^ Abteilung für Allergologie und Immunologie Klink für Dermatologie Venerologie und Allergologie Charité – Universitätsmedizin Berlin

Sehr geehrte Herausgeber,

Allergische Reaktionen auf Epoxidharze äußern sich typischerweise als allergische Kontaktdermatitis oder aerogene Kontaktdermatitis.^1^, ^2^ Allergische Reaktionen vom Soforttyp sind hingegen selten, wurden jedoch in Einzelfällen beschrieben.^3^

Wir berichten über einen 43‐jährigen Patienten, der sich zur allergologischen Abklärung vorstellte, nachdem es während einer Wurzelkanalbehandlung zu einer generalisierten Urtikaria, einem Lippenödem, Engegefühl im Hals und Atemnot gekommen war. Die anaphylaktische Reaktion wurde notfallmäßig behandelt. Während der zahnärztlichen Behandlung wurden ein Lokalanästhetikum mit Articain sowie AH Plus Jet® Paste eingesetzt – ein Wurzelkanalfüllmaterial auf Basis eines Epoxid‐Amin‐Polymers, das Bisphenol A‐Diglycidylether enthält.

Der Patient hatte bereits bei früheren zahnärztlichen Behandlungen ähnliche, jedoch mildere Episoden erlebt. Zudem sei es im beruflichen Kontext zu Hautkontakt mit Epoxidharzen vor etwa 20 Jahren gekommen, bei dem juckende Hautveränderungen auftraten. Obwohl er aufgrund dessen den Beruf gewechselt hat, wurde zu dieser Zeit keine allergologische Abklärung durchgeführt. Die Anamnese ergab ein kindliches Asthma bronchiale und eine arterielle Hypertonie, die aktuell mit Candesartan behandelt wird.

Die allergologische Abklärung umfasste einen Pricktest mit verschiedenen Lokalanästhetika (Procain, Lidocain, Bupivacain, Prilocain, Articain, Mepivacain), der negativ ausfiel. Zusätzlich wurde ein 20‐minütiger Epikutantest mit Epoxidharz (auf Basis von Bisphenol‐A‐Diglycidylether) aus der Standardreihe sowie der Kunstharze/Kleber‐Reihe der *Deutschen Kontaktallergiegruppe* durchgeführt. Der Test führte zu einer ausgeprägten urtikariellen Hautreaktion an den Teststellen für Epoxidharz, 1,4‐Butandiol‐Diglycidylether, 1,6‐Hexandiol‐Diglycidylether und Butylglycidylether (Abbildung 1). Spätreaktionen traten nicht auf.

**ABBILDUNG 1:**
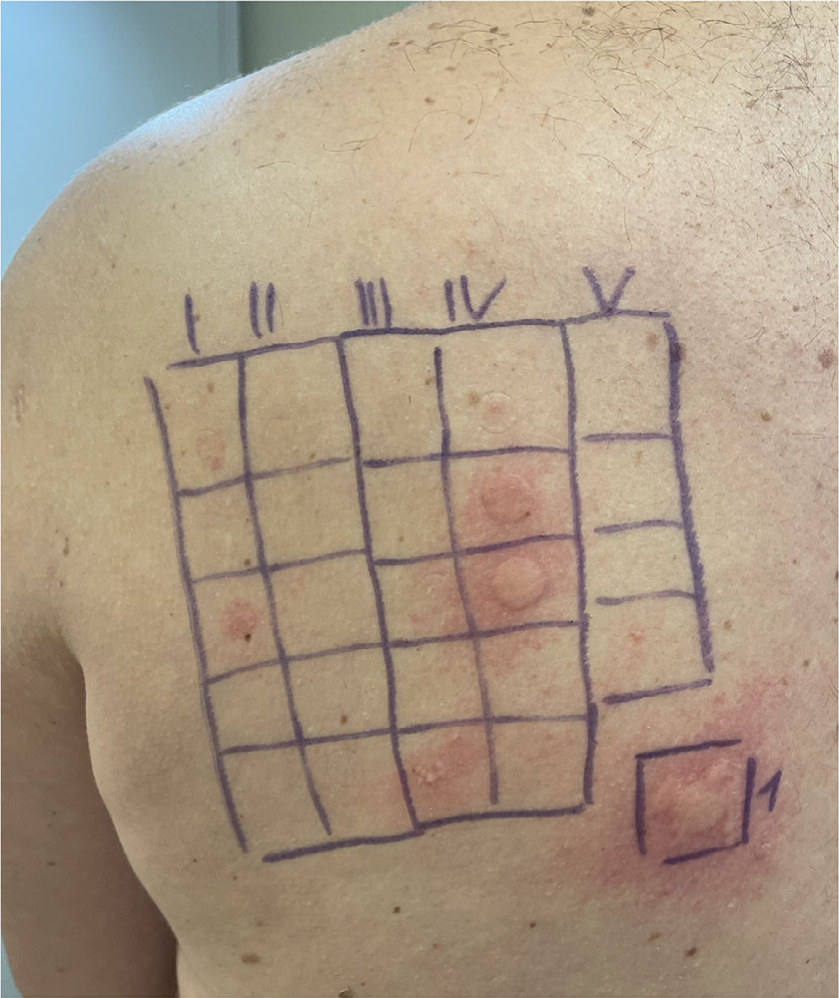
Positive Sofortreaktionen im Epikutantest auf Epoxidharz (Nummer 1), Butylglycidylether (Reihe III, Position fünf), 1,4‐Butandiol‐Diglycidylether (Reihe IV, Position zwei) und 1,6‐Hexandiol‐Diglycidylether (Reihe IV, Position drei). Das Erythem im Bereich der ersten Reihe (Position eins und drei) wurde als irritative Reaktion gewertet.

Ein Pricktest auf inhalative Allergene zeigte positive Reaktionen auf Birke, Gräser und Beifuß. Der Gesamt‐IgE‐Wert im Serum war mit 229 kU/l erhöht. Spezifisches IgE gegen Latex war negativ, das Differenzialblutbild war unauffällig.

Wir konnten Epoxidharz auf Basis von Bisphenol‐A‐Diglycidylether als Auslöser der anaphylaktischen Reaktion identifizieren. Epoxidharze finden in zahlreichen Bereichen breite Anwendung, unter anderem in Klebstoffen, Kunststoffen, Bodenbeschichtungen, Isoliermaterialien, Farben, Lacken und Zahnfüllungen. Der Begriff „Epoxidharz“ bezeichnet in der Regel ein Harzsystem, das aus dem eigentlichen Harz, einem Härter, reaktiven Verdünnern sowie weiteren Zusatzstoffen besteht – sämtliche mit potenziell allergenem Charakter.^4^, ^5^ Etwa 75 % der heute eingesetzten Epoxidharze basieren auf Bisphenol‐A‐Diglycidylether (BADGE), das zugleich als der stärkste Sensibilisator gilt.^6^ Aufgrund seiner weiten Verbreitung stellt Epoxidharz ein zunehmend relevantes Kontaktallergen dar, wobei sich allergische Reaktionen vor allem in Form von Kontaktdermatitis oder aerogener Kontaktdermatitis manifestieren.^1^, ^2^, ^6^

Die Sensibilisierung gegenüber Epoxidharzen erfolgt zumeist berufsbedingt – typischerweise durch direkten Hautkontakt, aerogene Exposition, kontaminierte Materialien, unzureichende Schutzmaßnahmen oder den Umgang mit nicht vollständig ausgehärtetem Epoxidharz.^7^ Ausgehärtete Epoxidharze gelten hingegen als unbedenklich, da sie weder sensibilisierend wirken noch allergische Reaktionen auslösen.^5^

Im Gegensatz zur Typ‐IV‐Sensibilisierung, die typischerweise Ekzeme verursacht, sind bislang nur wenige Fälle von Typ‐I‐Allergien gegenüber Epoxidharzsystemen beschrieben worden. Diese äußern sich meist in Form einer Kontakturtikaria, seltener auch als allergische Rhinitis oder beruflich bedingtes Asthma bronchiale.^3^, ^4^, ^8^ Bisher ist in der Literatur nur ein einziger Fall einer anaphylaktischen Reaktion auf Epoxidharz dokumentiert.^9^ Nach unserem Kenntnisstand handelt es sich bei dem vorliegenden Fall um die zweite beschriebene Anaphylaxie durch Epoxidharz auf Basis von Bisphenol‐A‐Diglycidylether. Auch im Anaphylaxie‐Register sind bislang keine entsprechenden Fälle gemeldet worden.

Die Diagnose wurde in Zusammenschau des klinischen Beschwerdebildes und der positiven Reaktion im 20‐minütigen Epikutantest mit Auftreten einer Kontakturtikaria als Hinweis einer IgE‐vermittelten Reaktion gestellt. Aufgrund der schweren behandlungsbedürftigen systemischen Reaktion nach Exposition, der urtikariellen Reaktion im Epikutantest sowie der fehlenden Verfügbarkeit einzelner Testsubstanzen wurde auf weiterführende Testungen verzichtet.

Wie bereits in der Erstbeschreibung vermutet,^9^ war auch in unserem Fall der direkte Kontakt des Allergens mit der Schleimhaut im Rahmen der zahnärztlichen Behandlung bei einem hochsensibilisierten Patienten Auslöser der anaphylaktischen Reaktion. Die primäre Sensibilisierung erfolgte vermutlich im beruflichen Kontext, als der Patient nach Kontakt mit Epoxidharz erstmals juckende Hautveränderungen entwickelt hat.

Der vorliegende Fall verdeutlicht die Notwendigkeit, auch seltene Allergene wie Epoxidharze bei der differenzialdiagnostischen Abklärung von Anaphylaxien in Betracht zu ziehen – insbesondere bei entsprechender beruflicher Vorgeschichte und anamnestischen Hinweisen auf vorangegangene allergische Reaktionen. Laut Daten des Anaphylaxie‐Registers zählen Insektenstiche zu den häufigsten Ursachen berufsbedingter Anaphylaxien, gefolgt von Nahrungsmitteln und Medikamenten.^10^

Bekannte Typ‐IV‐Allergene (unter anderem Duft‐ und Konservierungsstoffe) können jedoch bei einem Teil der Sensibilisierten auch Reaktionen vom Soforttyp auslösen.^11^ Daher sollte bei Verdacht auf eine Soforttypreaktion aufgrund der Anamnese beim Epikutantest eine Ablesung nach 20 Minuten erfolgen und bei Auftreten stark positiver Reaktionen abgebrochen werden.

## DANKSAGUNG

Open access Veröffentlichung ermöglicht und organisiert durch Projekt DEAL.

## INTERESSENKONFLIKT

Keiner.
